# Cross-Sectional Investigation of Stress Fractures in German Elite Triathletes

**DOI:** 10.3390/sports7040088

**Published:** 2019-04-15

**Authors:** Pauline Neidel, Petra Wolfram, Thilo Hotfiel, Martin Engelhardt, Rainer Koch, Geoffrey Lee, Stefan Zwingenberger

**Affiliations:** 1 Department of Sports Medicine at the University Center for Orthopaedics and Traumatology, University Medicine Carl Gustav Carus, Technical University Dresden, 01307 Dresden, Germany; Stefan.Zwingenberger@uniklinikum-dresden.de; 2 Department for Sports Science, German Triathlon Federation, 60528 Frankfurt/Main, Germany; wolfram.petra@googlemail.com; 3 Department of Orthopaedic Surgery, Friedrich-Alexander-University Erlangen-Nuremberg, 91054 Erlangen, Germany; Thilo.Hotfiel@fau.de; 4 Department of Trauma and Orthopedic Surgery, Klinikum Osnabrück, 49076 Osnabrück, Germany; Martin.Engelhardt@klinikum-os.de; 5 Department of Medical Statistics and Biometry, Medical Faculty Carl Gustav Carus at Technical University Dresden, 01307 Dresden, Germany; rainer.koch.01@gmx.de; 6 Kennedy Institute of Rheumatology, Nuffield Department of Orthopaedics, Rheumatology, and Musculoskeletal Sciences, University of Oxford, Oxford OX3 7FY, UK; geoffrey.lee@kennedy.ox.ac.uk

**Keywords:** triathlon, injury, stress fracture, risk factors, prevention

## Abstract

Triathlon is a popular sport for both recreational and competitive athletes. This study investigated the rates and patterns of stress fractures in the German national triathlon squad. We developed a web-based retrospective questionnaire containing questions about the frequency of stress fractures, anatomic localisation and associated risk factors. The survey was conducted as an explorative cross-sectional study. Eighty-six athletes completed the questionnaire. Twenty athletes (23%) sustained at least one stress fracture. All documented stress fractures were located in the lower extremities. Factors associated with a higher risk for stress fractures were female gender, competitive sport prior to triathlon career, Vitamin D or iron deficiency, menstrual disturbances and a high number of annual training hours. Disseminating knowledge among athletes and their professional community in order to raise awareness about early symptoms and relevant risk factors could help to improve prevention and reduce the incidence of stress fractures.

## 1. Introduction

Triathlon is an endurance sport that is growing in popularity. Given its multisport nature, it comprises three disciplines in one event: swimming, cycling, and running. There are numerous competitive events ranging from sprint distance (0.75 km–20 km–5 km) to long distance triathlon (3.8 km–180 km–42 km). Moreover, related formats are duathlon or relay competitions. The Olympic Games reflects the highest achievable sporting event for short distance athletes. Triathlon has been included in the Olympic Program since Sydney 2000. Among long distance triathletes, the Ironman^®^ World Championships Hawaii, represents the most famous competition. 

In Germany, triathlon is governed by the Deutsche Triathlon Union (DTU). Currently, DTU includes 58,000 members, and continues to grow [1]. An important function of the DTU is the development and promotion of young and elite athletes. These are organized in a system of different national and federal state squads [2]. In order to provide a structured training environment, DTU has set up several national training bases where most of the squad athletes are located [2]. 

Injuries and the consecutive loss of training and cancellation of competitions inevitably have an impact on athletes. Various studies showed that in triathlon, overuse injuries are more common than acute injuries, accounting for up to 79% [3,4,5,6]. Recent observations of DTU confirm the rising incidence of stress fractures among their squad athletes [7]. Furthermore, the latest cases of stress fractures in professional long-distance triathletes like Jan Frodeno and Ben Hoffmann, forcing them to default the World Championships in Hawaii, show the devastating consequences of this injury [8,9]. Consequently, the need to identify potential risk factors led to the conception of this study. Another aspect is that, to our knowledge, there is no investigation about the occurrence of stress fractures in triathletes. In order to improve the prevention of stress fractures and the management of risk factors, it is crucial to understand which athletes are prone to this injury. Therefore, data about the occurrence of stress fractures and their anatomic localisation, as well as relevant factors that are aetiologically associated with stress fractures, were requested. The primary aim of this study was to identify the prevalence of stress fractures among German elite-triathletes. The secondary aim was to find factors that may act as possible predictors of stress fractures such as gender, BMI, triathlon career, nutritional deficiencies, menstrual irregularities or biomechanical factors. This could lead to new hypotheses and further studies to find causal relationships.

## 2. Materials and Methods

This investigation was carried out as an explorative cross-sectional study using a web-based questionnaire. In November 2017, athletes of the 2017 DTU-Triathlon squad were contacted by email either directly or indirectly via their national coach. Beforehand, all national coaches were introduced to the study during a DTU Elite Sport Conference. They received both a description of the study for informed consent and an online link with access to the web-based questionnaire. Athletes were then asked to complete the questionnaire on a voluntary basis. Participants gave their informed consent by completing the questionnaire. In January 2018, the survey period was closed.

The study particularly focused on elite athletes. Therefore, a precondition for participation was the membership to a German national squad (A-, B- or C-team) or Federal State squad (D- or D/C-team). In 2017, the German national team involved 59 athletes. Distributed over the 16 state federations; the Federal State squads included 209 athletes. Consequently, 268 athletes were potentially available and contacted for this investigation. 

All information was collected retrospectively using both open-ended and multiple choice questions. The questionnaire was distributed into three sections: 1. General questions about anthropometric information, e.g., age, gender or body weight; 2. Questions with regard to their training habits and health issues such as menstrual function or nutritional status; 3. Questions about the occurrence of stress fractures divided into the time before, during and after the fracture. Athletes who had not yet sustained a stress fracture were allowed to exit the questionnaire after finishing the second section. All information was gathered by laypeople. 

After the survey period, all data were collated into Microsoft Excel^®^ (Version 15.0; Microsoft Corporation, Redmond, WA, USA) and anonymised. Subsequently, data were transferred to SAS^®^ (Version 9.4; SAS Institute, Cary, NC, USA) for statistical analysis. For the purpose of data description, the results were specified by median (Q_1_; Q_3_), average (±SD) and minima and maxima. The central tool for the measurement of risk was univariate Odds Ratio (OR), followed by a test for significance using Pearson’s chi-square-test, Fisher’s exact test (when sample size was low) or Wald-tests (for continuous variables). Univariate ORs were estimated using an SAS-Macro which was based on Wald confidence limits. For all tests, the significance level was set to 5%. The calculation of the incidence rate was performed as division of the sum of the number of stress fractures with the sum of total person-years under risk. Due to the chosen study design, that is cross-sectional investigation, and due to the high number of targeted events, ORs should not be interpreted as measurements of relative risks.

All procedures were carried out in accordance with the local ethics committee of Technical University Dresden (Protocol number: EK 399102017; Date of approval: 26 September 2017) as well as with the Declaration of Helsinki. 

## 3. Results

### 3.1. All Survey Participants

A total of 136 athletes (50.7%) responded to the survey. Fifty athletes did not complete the questionnaire in its entirety. They mostly left the Online-survey in the first part (General questions) right after the beginning of the survey. Therefore, they were excluded from analysis due to incomplete data. Consequently, 86 (63.2%) athletes could be subjected to analysis. Of the 86 participants, 47 were male (55%) and 39 were female (45%) athletes. The anthropometric characteristics and training habits are shown in Table 1. On average, the athletes were 17.45 years old when they submitted the questionnaire. According to the age group definitions of DTU [10], the age group “Under 18” was the highest proportion. The distribution of athletes to the different squads is shown in Table 2 following the definitions of the German squad system until 2017 [11].

Of all participants, 56 (65%) had competed in another sport prior to their triathlon career. The most frequent sports were swimming (36%) and track and field sports (29%). All athletes participated mainly over short distances like Super Sprint (<0.5 km, <13 km, <3.5 km) and Sprint distance (0.75 km–20 km–5 km). As shown in Table 3, some athletes suffer from biomechanical issues, nutritional deficits or, referring to the female athletes, from menstrual irregularities. Treatment of the biomechanical issues was mainly carried out by specific shoe inserts or physical therapy. Amongst the nutritional supplementation, Vitamin D and iron supplements were most frequently taken.

### 3.2. Injured Participants

At the time of submission, 24 athletes had sustained a stress fracture. Yet, only 20 responses could be statistically analysed because four participants reported self-diagnosed stress fractures or different injuries. Those replies had to be sorted out due to the low validity of self-reported stress fractures [12]. Hence, 15 athletes with one stress fracture, four athletes with two stress fractures, one athlete with three stress fractures and 66 athletes with no stress fracture were transferred into the evaluation. This totaled 23%. Taking the number of occurrences per total person-years into consideration, the incidence rate was 4.8%/year (95% CI (approximate confidence interval: 3.0–6.5%). Among the group of injured athletes, 8 (40%) were male and 12 (60%) were female athletes. Their detailed description can be found in Table 1 and Table 3. Seven athletes belonged to the German national squad and 13 athletes to a Federal State squad, especially to the D-Team. Fifteen athletes (75%) were active in a different competitive sport prior to their triathlon career. Common sports were swimming (N (%): 7 (47%)) and track and field sports (N (%): 7 (47%)). The number of biomechanical issues, nutritional deficiencies and menstrual irregularities can be found in Table 3.

All stress fractures were localised in the lower extremities. The main anatomic localisations were the metatarsal bones (52%). In four cases (19%), athletes sustained a fracture of the femur or femoral neck, respectively. In six cases, the lower leg was affected—2 times (10%) for each tibia, fibula and calcaneus. Athletes sustained their first reported stress fracture with an average age of 16.9 (±2.5) years. Minimum age was 12 years and maximum age was 22 years.

Prior to the occurrence of the stress fracture, 12 athletes (57%) had increased their training. This mainly (75%) referred to an increase in the distance per week, especially of the running distance. Furthermore, it seems plausible that 10 athletes (48%) had participated in a training camp for 10.45 (±3.50) days on average. However, changes of diet, body weight, equipment or technique played a minimal role. Likewise, a predominant hard surface or a specific running technique did not influence the injury occurrence. 

All athletes felt pain in the particular location as initial symptom. For the most part, the pain arose in training N (%): 16 (76%)), during (N (%): 19 (90%)) or after (N (%): 16 (76%)) activity and was increasing (N (%): 16 (70%)) or was consistent (N (%): 5 (22%)) in the course of time, respectively. However, regardless of the anatomic localisation, all symptoms were assigned to running. There was only one exception, namely a hip fracture which was noticed in swimming although the specific athlete reported to have felt the pain during the pushing-off movements off the wall.

The median value from the onset of symptoms until medical consultation was seven days (Q_1_: 3; Q_3_: 15). Further, between the first medical consultation and the time of diagnosis passed a median of three days (Q_1_: 1; Q_3_: 7) with a range of 0–150 days. X-ray (N (%): 13 (62%)) and magnetic resonance tomography (MRI) (N (%): 12 (57%)) were mainly used as diagnostic tools. Computer tomography was only used in three cases (13%). 

The vast majority (N (%): 17 (81%)) received conservative treatment. However, in two cases athletes had to undergo surgery: firstly, a stress fracture of the metatarsals and secondly, a stress fracture of the tibia. The median value of training downtime in terms of rehabilitation was 70 days (Q_1_: 28; Q_3_: 120) with a wide range of 6–180 days. The longest break (120 days; Q_1_: 110; Q_3_: 135) was seen in stress fractures of the femur, followed by stress fractures of the tibia (69 days; Q_1_: 49; Q_3_: 90) and calcaneus (60 days; Q_1_: 55; Q_3_: 65). In contrast, the shortest break in training was caused by stress fractures of the metatarsal bones (50 days; Q_1_: 28; Q_3_: 120) and fibula (55 days; Q_1_: 48; Q_3_: 63). While training was interrupted in the affected discipline, most of the athletes trained in alternative sports such as swimming (N (%): 14 (78%)) and biking (N (%): 11 (61%)). Taking all athletes with stress fractures into consideration, the period from symptom onset until return to training amounted to 80 days, disregarding the fact that there may have been temporal overlaps.

### 3.3. Risk Factors

The following table (Table 4) shows the results of the analysis of univariate Odds Ratio (OR):

## 4. Discussion

A central aim of this investigation was the identification of factors that may be associated with an increase of risk for stress fractures. This is of importance because identifying factors that place athletes at greater risk for injury can be crucial to reduce the occurrence of injuries [4]. Hence, this knowledge can help to improve prevention and knowledge about stress fractures and can also lead to new hypotheses to find causal relationships.

Taking all aspects into consideration, among German elite triathletes, athletes with the following factors were associated with sustaining a stress fracture: female gender, background in competitive sports, Vitamin D or iron deficiency, nutritional supplementation, irregular menstrual cycles and high total training amounts. 

When projected on an entire year, the duration of training amounted to 853 h per year. In contrast, the national guidelines for adolescent athletes of age group “Jugend A” only recommends 700 h training per year [13]. Therefore, the participating athletes exceeded the standard requirements. 

The main observations including practical implications were as follows: The majority of the recorded stress fractures arose after an increase of training which may be associated with attending a training camp. The first related sign was pain during or after running training. After one week, a physician was consulted, who could enable a prompt diagnosis with the aid of radiological imaging. Conservative treatment was the predominant course and alternative sports were trained in the 70-day training downtime. 

This study is the first that has explored stress fractures exclusively in the unique population of elite triathletes. Comparative information can therefore only be determined by studies about cross and field athletes or athletes in general (i.e., Bennell et al. [14] or Changstrom et al. [15]) or studies about overuse injuries in triathlon (i.e., Collins et al. [16], Korkia et al. [3], Burns et al. [4] or Vleck et al. [6]).

The athletes surveyed were predominantly of the younger generations (average age: 17.45 years) who were restricted to starting on the shorter distances according to the regulations of DTU [10]. At this point of their career, the adolescent athletes place particular emphasis on the substantial increase of training volume in order to improve their physical stress tolerance [13,17]. However, in younger generations the full extent of training volume is not yet reached [17]. 

In comparison with other studies, the average age in the present study was clearly younger [3,4,6,18]. Furthermore, the participants did not include amateur athletes, but rather ambitious athletes that execute triathlon professionally and had to verify their status as squad members through performance tests or competition results [2]. In the majority of previous studies, no distinction in terms of professionalism was made.

Various studies showed that triathletes were rather prone to overuse injures than to acute trauma [5]. A key research issue was to investigate the frequency and incidence of stress fractures among professional triathletes. In the present survey, 20 athletes (23%) already sustained a stress fracture prior to the survey period. A reason for this relatively high amount of stress fractures may be the low response rate or an over-representation due to selection bias [19,20]. The total incidence rate per year across all athletes was 4.8% (95% CI: 3.0–6.5%). In comparison with other studies that found incidence rates of 6.5–9.7% among athletes of different sports, this number is relatively low [21]. More precisely, runners and gymnasts show the highest incidence rates, whereas the lowest are found in swimming and diving [15,22,23]. These assumptions are relevant in triathlon since running training is given a high priority. Even though swim training requires a high proportion of the total training volume as well, it plays a minor role in injury aetiology [3]. 

In this study, as in many others, all stress fractures were located in the lower extremities [3,4,24,25]. Even further, McHardy et al. [5] showed that the lower extremities are the most affected anatomic site for all kinds of injuries in triathlon. In the context of overuse injuries specifically, the lower extremities are affected in 72–75% of cases [4]. Compared to cross and field athletes and runners, 61–78% of the stress fractures were also mainly located in the lower extremities [15,26]. 

Therapeutic decisions are often based on the common classification into high- and low-risk fractures depending on anatomic localisation [26,27]. Owing to the methodical approach to implement a questionnaire with laypeople, precise anatomic localisation cannot be described. For this reason, a clear classification into high- and low-fractures is needed.

Most previous studies agree that the aetiology appears multifactorial [25,26,28]. Accordingly, stress fractures evolve when different intrinsic and extrinsic components come together and frequently in combination with changes in training volume or intensity [4,5,6,26,29]. The multiple different risk factors are the subject of controversial discussion. However, it is the general consensus that women are at greater risk for stress fractures than men [14,15,23,30]. This corresponds to the findings of this study and to Fredericson et al. [31] who found a 1.5–3-times higher risk of females developing stress fractures. 

The “Female Athlete Triad (FAT)” comprises eating disorders, menstrual disturbances and low bone density and represents an underlying pathomechanism [32]. Correspondingly, the FAT was also seen in the present study: ten out of 17 (59%) of the female athletes who suffer from menstrual disturbances also sustained a stress fracture. Moreover, of the 12 female athletes with stress fractures, only two did not show menstrual irregularities. According to Bennell et al. [33], amenorrhoeic athletes have a higher stress fracture rate and a 2–4 times greater risk than athletes with regular menses. 

Further development led to a more complex definition and understanding of the FAT, namely the Relative Energy Deficiency in Sport (RED-S) [34]. RED-S describes a syndrome which influences a broad range of physiological functions (i.e., metabolic rate, menstrual function, bone health or immunity), all initially resulting from a relative energy deficiency [34]. Consequently, RED-S may lead to serious short-term and long-term effects on optimal health and performance, including an increased risk for stress fractures [34]. Besides, a major difference between FAT and RED-S is that men can be affected as well. 

In sports such as running or triathlon, where athletes may benefit from a low BMI/body weight, athletes are likely to be more prone to RED-S [26]. Despite the fact that in our survey the average BMI (19.9 kg/m^2^ (±1.4); Min/Max: 16/24) was within the normal range, they can still show inadequate intake of nutrients and energy. In this context, the exposed nutritional deficiencies as well as the low BMI of the athlete with three stress fractures could indicate an insufficient energy intake. Nevertheless, in previous studies a relation between BMI and the occurrence of stress fractures could not be clearly identified [35,36]. Even though body composition cannot be associated as a risk factor for stress fractures, the surveillance of BMI and its individual trend is still a helpful clinical tool to monitor health and nutritional status [33]. 

Nearly all studies agree that running is a major risk factor for triathlon-related injuries [3,4,6,16,24,25,33,37]. Thus, a high or increasing running mileage in preparation leads to an increased injury incidence [4,31]. Although athletes with stress fractures completed a higher distance in running training than their fellow athletes without stress fractures, an increase of OR could not be found. Nevertheless, all athletes took first notice of related symptoms during running. However, even more important in injury aetiology is less the total amount of training, but rather rapid changes in training programme without adequate time for adaptation [3,26,31]. In the present study, half of the athletes (52%) stated to have increased their training amount prior to the occurrence of the injury. Some athletes (46%) had even attended a training camp, where training mileage is typically boosted. Therefore, this seems to be a plausible pathomechanism in the study population. 

In competitive sports, the prevention of injuries, along with short healing time, is by far the most important aim of sports medicine [14]. The multifactorial aetiology plays a key role in prevention work. Due to this multifactorial nature, stress fractures cannot be traced back only to training manners but likewise, they cannot be prevented only by training management. Indeed, the injury aetiology is understood as an interaction of several different individual factors. For this purpose, risk factors such as Vitamin D deficiency and menstrual disturbances should be subject to targeted monitoring and diagnostics. In the case of positive diagnostic tests, they should obviously also be targeted for treatment. At the same time, attention should be drawn towards an adequate energy intake to avoid the emergence of RED-S [34]. Whenever there is evidence for the assumption that RED-S or menstrual disturbances exist, energy intake should be increased and/or physical activity should be reduced [34].

Increases or changes in training, especially in running, i.e., in training camps, should be carried out gradually and with sufficient time for adaptation. Including low-impact sports and discharging days could be an approach to reduce physical load [36,37]. 

Stress fractures occur along a continuum from stress reactions to eventually stress fractures [33]. This continuous progress also includes the chance to break the cycle by identifying early symptoms such as a load-dependent pain in the lower extremities, followed by a period of reduced activity [33]. As a result, healing time and time off training may be reduced. Since athletes often underestimate the severity of symptoms, they should be made aware of the importance of early diagnosis particularly [25,38]. In the end, cooperation between sport physicians, physiotherapists, coaches and athletes is crucial to enhance prevention work. 

Treatment after the occurrence of stress fractures should always be controlled by symptoms and must be continued until complete freedom of pain [26,39]. Simultaneously, risk factors need to be addressed in order to avoid further fractures [23]. 

The major limitations of this investigation are the limited measurement of exposure time, selection bias as a result of the voluntary participation, missing data of Non-Responders and the study design (cross-sectional study). It must be argued that cross-sectional studies cannot reveal causal relationships but only observed relationships. However, it is suitable to reveal new hypotheses and to identify potential risk factors. Furthermore, similar strategies were also adopted in other studies [3,5,15,18,28]. Carrying out a prospective cohort study with adequate power could explore causality in future studies. 

The different exposure times of athletes were combined with the sum of person-years under risk in the denominator of the calculation of incidence rate. An individual approach or a differentiation to specific phases of training were not performed. In addition, due to the relatively low number of participants, there are only a few statistically significant results. Furthermore, carrying out multivariate analyses did not provide reasonable models. Thus, a sufficient statistic model could not be developed and confounder-associated estimations of risk were impossible.

The survey was based on information of medical amateurs (including nutritional deficiencies and misalignment of feet and leg axis) in spite of the limited validity of self-reported injuries [12,40]. Another limitation is that no standardised methods and definitions were applied to all studies [5,6,41]. For this reason, a comparison of study results may be problematic. 

In order to enhance reliability and significance, the results need to be reexamined by studies with a larger sample size. All aspects and results need to be interpreted with consideration of the presented limitations. 

## 5. Conclusions

Stress fractures in elite triathletes are a common injury and are highly relevant. It has been shown that running is closely linked to injury aetiology. Likewise, fracture localisations were similar to those seen in runners. Various factors could be assigned to the occurrence of stress fractures which underlines the multifactorial aetiology. In order to disseminate knowledge about relevant risk factor and typical disease progressions athletes, coaches, physiotherapists and sports physicians should be educated. By addressing potential risk factors and adjustment of training strategies especially in running, prevention may be improved and downtimes in training reduced. Future studies should be designed to identify causes of stress fractures. 

## Figures and Tables

**Table 1:** Anthropometric characteristics, triathlon career, weekly training habits and the number of competitions of all participants, the group of athletes who sustained a stress fracture and of those who did not sustain a stress fracture are listed. All numbers refer to the 2017 season due to the retrospective nature of the survey. For all categories, average values (±SD), minima and maxima are shown.

Categories	All Participants	Injured Participants	Non-Injured Participants
	Average (±SD)—Min/Max	Average (±SD)—Min/Max	Average (±SD)—Min/Max
**Anthropometric characteristics**			
Age (years)	17.5 (±3.3)—12/34	18.0 (±2.7)—12/23	17.3 (±3.4)—13/34
BMI (kg/m^2^)	19.9 (±1.4)—16/24	20.4 (±1.9)—16/24	19.8 (±1.2)—16/22
**Triathlon career**			
Experience in triathlon (years)	6.4 (±2.8)—1/14	5.9 (±2.7)—3/11	6.5 (±2.9)—1/14
**Weekly training habits**			
Duration/week, Total (h)	16.4 (±5.5)—7/46	19.5 (±8.0)—8/46	15.5 (±4.1)—7/25
Duration/week, Swim (h)	6.0 (±2.4)—0/20	7.2 (±3.4)—3/20	5.7 (±2.0)—0/10
Duration/week, Bike (h)	4.1 (±1.9)—0/10	5.1 (±2.1)—2/10	3.8 (±1.8)—0/8
Duration/week, Run (h)	3.9 (±1.7)—1/12	4.6 (±2.1)—3/12	3.7 (±1.6)—1/12
Duration/week, Others (h)	2.3 (±1.3)—0/6	2.7 (±1.5)—1/6	2.1 (±1.3)—0/6
Distance/week, Swim (km)	16.3 (±5.6)—1/40	19.5 (±6.4)—6/40	15.6 (±4.8)—1/25
Distance/week, Bike (km)	110.2 (±55.6)—4/241	131.6 (±57.2)—31/241	105.7 (±52.9)—0/240
Distance/week, Run (km)	34.6 (±13.6)—4/70	37.6 (±13.9)—7/70	34.2 (±13.0)—6/60
**Annual competitions**			
Competitions (no.)	8.4 (±3.2)—0/21	7.8 (±4.0)—0/15	8.7 (±3.0)—0/21

**Table 2:** The membership to either the German national squad or one of the 16 Federal State squads is shown. According to the squad system until 2017, squads are further divided into an A-, B- or C- team within the national squad and into a D/C- or D-team in the Federal State squad. For all categories, absolute and relative proportions are listed.

Categories		N (%) (n = 86)	Total (n = 86)
German national squad	A	1 (1%)	15 (17%)
	B	10 (12%)	
	C	4 (5%)	
Federal State squad	D/C	13 (15%)	71 (83%)
	D	58 (67%)	

**Table 3:** Categorical variables to describe gender distribution, biomechanical issues, nutritional deficits and menstrual irregularities. The numbers refer to the group of all athletes, injured and non-injured athletes. For all categories, numbers and percentages are shown.

Categories	All Participants	Injured Participants	Non-Injured Participants
	N (%) (n = 86)	N (%) (n = 20)	N (%) (n = 66)
**Gender**			
Female gender	39 (45%)	12 (60%)	27 (41%)
Male gender	47 (55%)	8 (40%)	39 (59%)
**Biomechanical issues**			
Misalignment of feet	27 (31%)	4 (20%)	23 (35%)
Misalignment of leg axis	14 (16%)	2 (10%)	12 (18%)
Unequal leg length	10 (12%)	1 (5%)	9 (14%)
Unspecified	3 (3%)	0	3 (5%)
Treatment ^1^	19 (35%)	3 (43%)	16 (24%)
**Nutritional deficits**			
Iron deficiency	29 (34%)	9 (45%)	20 (30%)
Vitamin D deficiency	10 (12%)	3 (15%)	7 (11%)
Nutritional supplementation	24 (28%)	9 (45%)	15 (23%)
**Menstrual irregularities ^2^**			
Irregular menstrual cycles	12 (31%)	5 (42%)	7 (11%)
Amenorrhea	5 (13%)	5 (42%)	0

^1^ Refers to those athletes who claimed a biomechanical issue. ^2^ Refers only to female athletes (n = 39).

**Table 4:** Categories with OR > 1 and OR < 1 are listed. ORs are given for each category as well as 95% confidence intervals and *p*-values. The calculation of the *p*-value was based on chi-squared tests and Wald tests with single or multiple fractures as event.

Category	OR	95% CI	*p*-Value
Female gender	2.2	0.8–6.0	0.1330
Competitive sport prior to triathlon	1.8	0.6–5.7	0.2898
Vitamin D deficiency	1.6	0.4–6.8	0.5612
Iron deficiency	2.2	0.7–6.6	0.1544
Nutritional supplementation	2.8	1.0–8.0	0.0517
Total amount of training ^2^	1.2	1.0–1.3	0.0173
Irregular menstrual cycles ^1^	1.7	0.4–7.4	0.4533
BMI ^2^	1.3	0.9–1.9	0.1541
Distance/week, Run ^2^	1.0	1.0–1.1	0.6080
Misalignment of feet	0.4	0.1–1.3	0.1008
Misalignment of leg axis	0.5	0.1–2.4	0.3732
Experience in triathlon/Years in triathlon ^2^	0.9	0.7–1.1	0.2872
Number of competitions ^2^	0.9	0.8–1.1	0.1990
Unequal leg length	0.3	0.0–2.5	0.2333

^1^: This category only applies to female athletes. ^2^: For quantitative variables, an increase of OR refers to an increase by one unit of measurement.

## References

[1] Deutsche Triathlon Union e.V Triathlon in Zahlen. https://www.dtu-info.de/triathlon-in-zahlen.html.

[2] Deutsche Triathlon Union e.V DTU Nominierungskriterien Kader. https://www.dtu-info.de/a/dateien/leistungssport/Nominierungskriterien/2018/DTU_Nominierungskriterien%20Kader%202017-2020.pdf.

[3] Korkia P.K., Tunstall-Pedoe D.S., Maffulli N. (1994). An epidemiological investigation of training and injury patterns in British triathletes. Br. J. Sports Med..

[4] Burns J., Keenan A.-M., Redmond A.C. (2003). Factors associated with triathlon-related overuse injuries. J. Orthop. Sports Phys. Ther..

[5] McHardy A., Pollard H., Fernandez M. (2006). Triathlon injuries: A review of the literature and discussion of potential injury mechanisms. Clin. Chiropr..

[6] Vleck V.E., Bentley D.J., Millet G.P., Cochrane T. (2010). Triathlon event distance specialization: Training and injury effects. J. Strength Cond. Res..

[7] Hotfiel T., Wolfram P., Engelhardt M. (2017). Unpublished Personal Communication—Steigende Anzahl von Stressfrakturen.

[8] Deutsche Presse Agentur Saison-Aus wegen Stressfraktur: Frodeno verpasst Ironman-WM auf Hawaii. http://www.spiegel.de/sport/sonst/ironman-wm-auf-hawaii-jan-frodeno-muss-teilnahme-absagen-a-1227680.html.

[9] Müller S. Ironman-WM 2018. Ben Hoffman muss Hawaii-Start Absagen. http://tri-mag.de//szene/ben-hoffman-muss-hawaii-start-absagen-146365.

[10] Deutsche Triathlon Union e.V Die Sportordnung der Deutschen Triathlon Union. https://www.dtu-info.de/a/dateien/regelwerk-ordnungen/Ordnungen/SpO_2018_V_1_2_sw.pdf.

[11] Bundesministerium des Innern, Deutscher Olympischer Sportbund, Sportministerkonferenz Konzept zur Neustrukturierung des Leistungssports und der Spitzensportförderung. https://www.bmi.bund.de/SharedDocs/downloads/DE/publikationen/themen/sport/sport-spitzensport-neustrukturierung.pdf?__blob=publicationFile&v=1.

[12] Øyen J., Torstveit M.K., Sundgot-Borgen J. (2009). Self-reported versus diagnosed stress fractures in Norwegian female elite athletes. J. Sports Sci. Med..

[13] Pöller S., Möller T. (2013). Rahmentrainingskonzeption Nachwuchs der Deutschen Triathlon Union.

[14] Bennell K.L., Malcolm S.A., Thomas S.A., Reid S.J., Brukner P.D., Ebeling P.R., Wark J.D. (1996). Risk factors for stress fractures in track and field athletes. A twelve-month prospective study. Am. J. Sports Med..

[15] Changstrom B.G., Brou L., Khodaee M., Braund C., Comstock R.D. (2015). Epidemiology of stress fracture injuries among US high school athletes, 2005–2006 through 2012–2013. Am. J. Sports Med..

[16] Collins K., Wagner M., Peterson K. (1989). Overuse injuries in triathletes. Am. J. Sports Med..

[17] Neumann G., Pfützner A., Hottenrott K. (2010). Das große Buch vom Triathlon.

[18] Zwingenberger S., Valladares R.D., Walther A., Beck H., Stiehler M., Kirschner S., Engelhardt M., Kasten P. (2014). An epidemiological investigation of training and injury patterns in triathletes. J. Sports Sci..

[19] Hernán M.A., Hernández-Díaz S., Robins J.M. (2004). A Structural approach to selection bias. Epidemiology.

[20] Hammer G.P., du Prel J.-B., Blettner M. (2009). Avoiding bias in observational studies. Dtsch. Aerzteblatt Online.

[21] Wentz L., Liu P.-Y., Haymes E., Ilich J.Z. (2011). Females have a greater incidence of stress fractures than males in both military and athletic populations: A systemic review. Mil. Med..

[22] Brüntrup J. Viele Läufer Bekommen Stressfrakturen. https://www.aerztezeitung.de/panorama/sport/sportmedizin/article/450731/viele-laeufer-bekommen-stressfrakturen.html.

[23] Moreira C.A., Bilezikian J.P. (2016). Stress fractures: Concepts and therapeutics. J. Clin. Endocrinol. Metab..

[24] Shaw T., Howat P., Trainor M., Maycock B. (2004). Training patterns and sports injuries in triathletes. J. Sci. Med. Sport.

[25] Cipriani D.J., Swartz J.D., Hodgson C.M. (1998). Triathlon and the multisport athlete. J. Orthop. Sports Phys. Ther..

[26] Hotfiel T., Lutter C., Heiß R. (2018). Stressreaktionen/-frakturen—Newsletter der Gesellschaft für Orthopädisch-Traumatologische Sportmedizin. GOTS.

[27] Patel D.S., Roth M., Kapil N. (2011). Stress fractures: Diagnosis, treatment, and prevention. Am. Family Phys..

[28] Duckham R.L., Brooke-Wavell K., Summers G.D., Cameron N., Peirce N. (2015). Stress fracture injury in female endurance athletes in the United Kingdom: A 12-month prospective study: Stress fractures in female endurance athletes. Scand. J. Med. Sci. Sports.

[29] Migliorini S. (2011). Risk factors and injury mechanism in triathlon. J. Hum. Sport Exerc..

[30] Rizzone K.H., Ackerman K.E., Roos K.G., Dompier T.P., Kerr Z.Y. (2017). The epidemiology of stress fractures in collegiate student-athletes, 2004–2005 through 2013–2014 academic years. J. Athl. Train..

[31] Fredericson M., Jennings F., Beaulieu C., Matheson G.O. (2006). Stress fractures in athletes. Top. Magn. Reson. Imaging.

[32] Koenig S.J., Toth A.P., Bosco J.A. (2008). Stress fractures and stress reactions of the diaphyseal femur in collegiate athletes: An analysis of 25 cases. Am. J. Orthop..

[33] Bennell K., Matheson G., Meeuwisse W., Brukner P. (1999). Risk factors for stress fractures. Sports Med..

[34] Mountjoy M., Sundgot-Borgen J., Burke L., Carter S., Constantini N., Lebrun C., Meyer N., Sherman R., Steffen K., Budgett R. (2014). The IOC consensus statement: Beyond the female athlete triad—Relative Energy Deficiency in Sport (RED-S). Br. J. Sports Med..

[35] Korpelainen R., Orava S., Karpakka J., Siira P., Hulkko A. (2001). Risk factors for recurrent stress fractures in athletes. Am. J. Sports Med..

[36] Field A., Gordon C., Pierce L., Ramappa A., Kocher M. (2011). Prospective study of physical activity and risk of developing a stress fracture among preadolescent and adolescent girls. Arch. Pediatr. Adolesc. Med..

[37] Spiker A.M., Dixit S., Cosgarea A.J. (2012). Triathlon: Running injuries. Sports Med. Arthrosc. Rev..

[38] Gosling C.M., Forbes A.B., Gabbe B.J. (2013). Health professionals’ perceptions of musculoskeletal injury and injury risk factors in Australian triathletes: A factor analysis. Phys. Ther. Sport.

[39] Larsen P., Elsoe R., Rathleff M.S. (2016). A case report of a completely displaced stress fracture of the femoral shaft in a middle-aged male athlete—A precursor of things to come?. Phys. Ther. Sport.

[40] Gabbe B.J., Finch C.F., Bennell K.L., Wajswelner H. (2003). How valid is a self reported 12 month sports injury history?. Br. J. Sports Med..

[41] Kienstra C.M., Asken T.R., Garcia J.D., Lara V., Best T.M. (2017). Triathlon injuries: Transitioning from prevalence to prediction and prevention. Curr. Sports Med. Rep..

